# Advanced Design and Implementation of a Biomimetic Humanoid Robotic Head Based on Vietnamese Anthropometry

**DOI:** 10.3390/biomimetics9090554

**Published:** 2024-09-15

**Authors:** Nguyen Minh Trieu, Nguyen Truong Thinh

**Affiliations:** Institute of Intelligent and Interactive Technologies, University of Economics Ho Chi Minh City—UEH, Ho Chi Minh City 70000, Vietnam

**Keywords:** Vietnamese anthropometry, anatomy, humanoid robot, emotional robot, geminoid robot, robotics

## Abstract

In today’s society, robots are increasingly being developed and playing an important role in many fields of industry. Combined with advances in artificial intelligence, sensors, and design principles, these robots are becoming smarter, more flexible, and especially capable of interacting more naturally with humans. In that context, a comprehensive humanoid robot with human-like actions and emotions has been designed to move flexibly like a human, performing movements to simulate the movements of the human neck and head so that the robot can interact with the surrounding environment. The mechanical design of the emotional humanoid robot head focuses on the natural and flexible movement of human electric motors, including flexible suitable connections, precise motors, and feedback signals. The feedback control parts, such as the neck, eyes, eyebrows, and mouth, are especially combined with artificial skin to create a human-like appearance. This study aims to contribute to the field of biomimetic humanoid robotics by developing a comprehensive design for a humanoid robot head with human-like actions and emotions, as well as evaluating the effectiveness of the motor and feedback control system in simulating human behavior and emotional expression, thereby enhancing natural interaction between robots and humans. Experimental results from the survey showed that the behavioral simulation rate reached 94.72%, and the emotional expression rate was 91.50%.

## 1. Introduction

Robotics is one of the most influential achievements in human life today, developed with advanced digital technology, giving robots more and more possibilities to serve various roles in life. Robotic technology has been identified as a potential tool to help older people live independently and is emerging as an innovative approach to supporting older adults directly, such as with robotic wheelchairs, and indirectly providing support to stakeholders, including caregivers [1]. Humanoid robots were developed to improve human–machine interactions in various fields, whereby humans and robots as colleagues could work together as collaborators to complete tasks in industrial environments. As a result, new human–robot interaction systems have been developed so that such systems can take advantage of the capabilities of both humans and robots [2]. Studies by psychologists show that only 7% of information is conveyed in spoken language, while 38% is expressed through tone of voice, and facial expressions convey 55% [3]. It can be seen that robots with facial expressions becoming integrated into human life will be an inevitable trend. 

Research on humanoid robots has advanced significantly in recent years. The concept of humanoid robots was first proposed in 1973 at Waseda University, with the WABOT-1 robot being regarded as the first complete humanoid robot in the world. Humanoid robots are simply understood as robots with the shape, size, and ability to perform functions similar to humans, including moving, grasping objects, and communicating [4]. One notable example is KISMET [5], a robot head developed in 1998 to simulate basic emotions using 17 degrees of freedom (DoF). KISMET features a cartoon-character-like face and a child-like voice. Following KISMET, numerous other humanoid robot heads have been introduced, including BARTHOC, HUBO, ROMAN, and ASIMO [6,7,8]. These robots are capable of expressing six to seven basic emotions and are designed using servo motors to generate these emotional expressions. Then, in the robotics laboratory at Osaka University, researchers introduced the first robotic geminoid, which was modeled by Professor Hiroshi Ishiguro, called HI–1 [9]. According to the author, geminoid robots represent a new branch in the definition of humanoid robots, designed and manufactured to resemble humans in both appearances and behavior. They include an artificial skin structure and anthropometric indicators [10]. Next, Geminoid F [11] is a female version of the Geminoid series, created to investigate the psychological and social aspects of human–robot interactions. Geminoid F can express fundamental emotions through facial movements and soft skin, allowing it to communicate naturally and convincingly. Then, there is Telenoid R1 [12], a robot with a simpler design and a genderless visage that can represent anyone in remote conversation. Telenoid R1 is utilized in studies of social and emotional interactions between humans and robots, particularly in healthcare and remote communication applications. Another robot, the Ibuki robot [13], was designed to resemble a child robot and can travel on wheels. It was created with the upper body in mind, focusing on mechanical processes in the wrist, arm, and head joints. With 15 degrees of freedom in the head, the robot can communicate basic emotions through facial expressions. Research on humanoid robots is increasingly popular and is applied in many different fields, such as medicine, education, health care, and customer service [14]. This study aims to propose an optimal design for a robot head based on the sustainable theories of medicine in human anatomy. In this study, the robot head is modeled according to the Vietnamese anthropometric dimensions, resulting in a close and user-friendly robot. This design focuses on both appearance and the capacity to accurately recreate emotions such as delight, surprise, grief, and rage. The robot’s prosthetic structures allow the robot to alter facial muscles softly and naturally, resulting in rich and varied expressions. In this study, a complete design for the humanoid robotic head is proposed based on anthropometrics, with a behavioral simulation rate of up to 94.72% and an emotional expression rate of 91.50%. The proposed structures are designed to ensure appropriate size while providing operating space to create realistic emotions.

This study proposes a comprehensive design for the biomimetic behavior of a robotic head based on Vietnamese anatomy and anthropometry. Section 2 presents the methodology including theoretical basics and dimensions for robotic design, which are used to create the robotic head. Section 3 presents robotic head designs, which were built based on fundamental theories to develop full mechanical designs that meet the angle limitations of facial parts. Finally, experiments were set up to evaluate the effectiveness presented in Section 4.

## 2. Materials and Methods

### 2.1. Theoretical Basics

Anthropometry is the science that studies the dimensions and proportions of the human body and applies them to product design to ensure fit, comfort, and efficiency for the user. Since ancient and medieval times, Egyptians used body proportions to build statues and structures and often followed precise proportions to create harmony and balance. Greek and Roman artists and architects studied the proportions of the human body to create works of art and architecture. In the 18th and 19th centuries, Johann Friedrich Blumenbach [15] was one of the pioneers in the field of anthropometry. Blumenbach classified humans into five main races based on morphological characteristics. Adolphe Quetelet [16] was a Belgian mathematician and statistician who applied statistical methods to the study of the human body. He developed the concept of the “average person” and body mass index (BMI). Francis Galton was an English researcher, who developed many methods of measuring and analyzing anthropometry [17]. He was also the founder of modern anthropometry and made important contributions to the fields of genetics and statistics. In the 20th century, anthropometric indices were applied in the military to design clothing and equipment to suit the different body sizes of soldiers and were widely applied in genetic research to better understand the differences and similarities between human groups. Anthropometry is the science that studies the dimensions and proportions of the human body and applies them to product design to ensure fit, comfort, and efficiency for the user.

**Figure 1:**
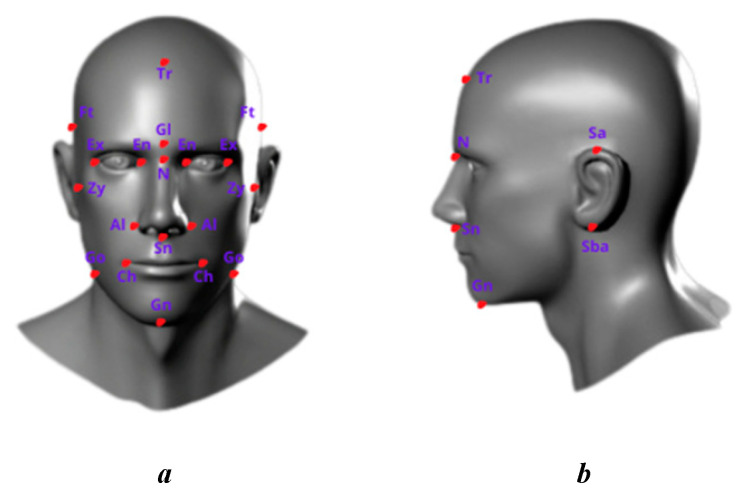
Locations and symbols of facial landmarks. (**a**) is a frontal view. (**b**) is a lateral view.

Accordingly, based on anthropometrics, the standards for the size and proportion of the robot head in terms of proportionality through indicators ensure that parts such as the neck, jaw, mouth, and eyes are set up and placed correctly. The right position creates a harmonious and natural feeling, thereby easily optimizing design and minimizing materials and production costs while still ensuring quality and functionality. From the standard size table, parameters related to the size and shape of the human head are used to design movement mechanisms, ensuring the robot can perform natural and harmonious movements like those of humans. It also helps determine ideal locations for sensors and cameras, ensuring the robot’s ability to observe and collect data effectively. In the design of humanoid robots, especially the head, the use of anthropometric indicators helps create a highly aesthetic product that is compatible with the dimensions and proportions of real humans. Therefore, anthropometric indices of the human head are examined in this study. The facial landmarks are defined following the theory of medical anthropometry, which is noted in Figure 1.

The process of determining the basic dimensions of a robot head requires selecting a sample group that is representative of the population the robot head will serve. This includes determining the age, gender, race, and other demographic factors. Precise measurement tools like calipers are then used to measure small distances like nose width and mouth length. Deep learning algorithms are used to extract the coordinates of the landmarks, which are presented in our previous work [18]. Next, measurements are taken from the selected objects, during which it is important to follow correct measuring techniques to ensure accuracy. Then, all data for each participant are recorded. After collecting enough data, analysis can begin to determine average values, standard deviations, and other statistical parameters that help determine standard sizes and tolerances for indicators. A 3D head model is proposed based on the anthropometric indicators determined according to the aesthetic dimensions in Table 1. The robotic head cannot be completely copied from a person due to personal information security concerns. The 3D model is checked to ensure all details are correct and consistent with previously analyzed and calculated values, and it is calibrated to achieve the desired results.

The three main views, including the front view, side view, and top view, are used to determine the size and parameters of the robot head. The front view shows the front of the robot’s head, allowing measurements of vertical and horizontal dimensions such as head length, head width, and the distance between facial points such as eyes, nose, and mouth. The side view provides information about the depth and lateral features of the head, including the projection of the nose and the position of the ears. The top view shows the horizontal balance of the head and helps accurately position the eye sockets and wings of the nose. A table of facial dimensions was created based on data collected from 203 people aged 18–36, of whom 125 were female (61.5%), with a mean age of 23.09 ± 12.32 years. This dataset is used and combined with an aesthetic ratio of the human face, as shown in Table 1, for the design of the humanoid robot. This data was collected in our previous study [18].

In anatomical analysis, researchers have provided a theoretical basis for the structure and function of important joints and muscles of the head and neck, which enable the human head to rotate and tilt due to the combination of joints, mainly the neck joints and the cervical spine. In particular, the atlanto-occipital joint (between the occipital bone and the first cervical vertebra) allows the head to nod up and down (flexion and extension). The atlantoaxial joint (between the first and second cervical vertebrae) allows the head to rotate left and right (rotation). The joints between the cervical vertebrae (C2–C7) support flexion/extension, lateral bending, and rotation [19]. The mandible is the only bone of the face that can move, connected to the skull through the temporomandibular joint (TMJ). This joint allows rotation and gliding movements, necessary for opening and closing the mouth and chewing. The main muscle bundles that control the jaw include the masseter, temporalis, medial pterygoid, and lateral pterygoid. These muscles work together to perform complex movements of the jaw. The upper jaw (maxilla) is a fixed part of the skull, unable to move independently. Eye movements are controlled by six extraocular muscles, including the superior rectus, inferior rectus, medial rectus, lateral rectus, superior oblique muscle (superior oblique), and inferior oblique muscle (inferior oblique). These muscles allow the eye to move in multiple directions and rotate within the orbit, protected by the orbital bones. Precise coordination between eye muscles allows the eyes to look in different directions and adjust focus flexibly. The combination of muscles, bones, and joints creates extremely complex human movements, especially emotions [20]. Anatomical investigation is essential to form the basis for designing robots that ensure the reproduction of the smallest movements of the robot. To create a robot capable of reproducing human emotions, the degrees of freedom of the robot must ensure at least the minimum DoF of the parts involved in creating human emotions. Data on moving muscle bundles and DoF of each part of the human face are examined in Table 2.

### 2.2. Dimensions for Robotic Design

After clearly understanding the size and number of degrees of freedom of the human head, choosing the size to draw the humanoid robot head becomes easier and more accurate. Firstly, the main dimensions of the human head, including width, height, and depth, as well as anthropometric distances such as distance between the eyes (interocular distance), length from the eyes, forehead-to-chin length, and distance between the ears (bi-auricular width), are determined. The degrees of freedom of the human head also need to be recreated to ensure the robot can perform natural movements such as turning, nodding, and tilting the head. To design the robot head, average anthropometric measurements were combined with aesthetic proportions to ensure aesthetics and compatibility with the human body. Parts such as the eyes, jaw, and neck will be designed according to corresponding degrees of freedom: the eyes have three degrees of freedom allowing movement in different viewing directions; the lower jaw has three degrees of freedom allowing opening, closing, and slight lateral movements; and the neck has six degrees of freedom to replicate the flexible movements of the head. The measurements used as the basis for the design of the humanoid robot are presented in Table 3.

### 2.3. Theory of Emotion Creation

Creating artificial human emotions is a burgeoning area of research that intersects the fields of artificial intelligence, cognitive science, and psychology. The biological theory of expression, derived from the work of Charles Darwin and further developed by Paul Ekman, posits that human facial expressions have a biological basis and are the result of evolution. According to this theory, facial expressions are not only a product of culture but are also universal across different cultures and species. Darwin argued that facial expressions evolved to serve a communicative function within the species, helping humans and animals communicate emotional states and intentions. Ekman, through cross-cultural research, identified seven basic emotions (happiness, sadness, fear, anger, surprise, disgust, and contempt) that are expressed through universal facial expressions. These expressions are controlled by the central nervous system, specifically the facial nerves and more than 40 facial muscles that coordinate to create complex expressions. According to Ekman, there are seven basic and universal emotions expressed through facial expressions: happiness, sadness, fear, anger, surprise, disgust, and contempt [21]. These expressions can be recognized and analyzed based on specific facial muscle movements, such as eyebrow-raising, lip pursing, and mouth opening. Designing humanoid robots with the capability to express and interpret human emotions is a complex and multidisciplinary endeavor. The design process involves not only sophisticated mechanical and electronic engineering to create lifelike movements and facial expressions but also advanced algorithms for emotion recognition and generation. Breazeal [3] underscores the importance of affective interaction in humanoid robotics, illustrating how emotional expression and recognition are crucial for the development of socially adept robots.

Emotions are divided into two main categories: primary emotions and secondary emotions. Primary emotions are natural and spontaneous responses to external stimuli and are often universal across cultures, such as joy, sadness, fear, and anger. They appear quickly and can be considered instinctive responses to specific situations. Meanwhile, secondary emotions are more complex and often involve thought processes and personal reflection. They include emotions such as shame, guilt, pride, and jealousy, which develop from a combination of primary emotions and personal and social experiences. Secondary emotions can vary across cultures and social contexts, and they often reflect human cognitive and social development. Ekman and Cordaro’s research presented a classification and understanding of primary and secondary emotions [22], emphasizing that this distinction helps us better understand how humans respond and interact with the world around them. Primary emotions are of particular interest in the investigation of robot emotion generation in this study.

## 3. Robotic Head Design

### 3.1. Neck Mechanism

The human neck is an important part of the body, connecting the head to the body and allowing for many flexible movements. This includes the bone structure of the cervical spine with seven vertebrae (C1 to C7), of which C1 (atlas) and C2 (axis) have a special structure that allows head rotation [23]. The atlanto-occipital joint provides the connection between the skull and C1, allowing for head nodding. The atlantoaxial joint connects C1 and C2, allowing head rotation. In short, the neck has three degrees of freedom, including three angular movements: nodding angle (flexion/extension), head tilt (lateral bending), and head rotation (rotation). To design a humanoid robot neck, the degrees of freedom and motion need to be replicated. This is done by using mechanical joints that simulate the cervical vertebrae, replacing them with motors to control the joints while also installing sensors to monitor the position and impact force. Light and durable materials such as aluminum or light alloys are used to ensure the robot neck is strong enough to support the head yet light for ease of movement. In this way, the robot neck can accurately simulate the functions and movements of the human neck, creating naturalness and flexibility in the robot’s operations. Accordingly, to easily and accurately simulate the functions and movements of the human neck, the parameters of the working space of the human neck are surveyed to propose the motor to reproduce the movements, as shown in Table 4.

Lateral bending does not directly contribute to the expression of core emotions, such as happiness, sadness, fear, and anger. These fundamental emotions are primarily represented through facial muscle movements involving the brows, eyes, mouth, and cheeks. Lateral bending is typically not required for the clear expression of these emotions. As a result, this degree of freedom is excluded from this study to simplify the neck load-bearing systems. The robot neck design with two degrees of freedom, including head nodding (flexion/extension) and head rotation (rotation), is proposed to achieve a balance between simplicity and basic expressive capabilities. In particular, the focus is on these two degrees of freedom reducing the complexity of the mechanical and control systems, thereby reducing costs and development time. A more limited number of joints and moving parts decreases the likelihood of problems and increases system durability, while specifically reducing the overall weight of the robot neck, making it lighter and easier to integrate into the overall robot design. In particular, nodding movements express emotions such as agreement, concentration, or sadness, and head rotation conveys behaviors such as looking around, paying attention, or showing interest in a specific direction, which is enough to reproduce many basic expressions and helps the robot interact effectively in many situations.

The robot neck design includes two main degrees of freedom: yaw and pitch. It uses a gear mechanism and lead screw to control movement. The gear mechanism facilitates the head rotation (yaw) movement. In particular, gear 1 is attached directly to the motor shaft. When the engine rotates, this gear also rotates. Gear 2 is locked to the middle shaft and meshes with gear 1. Gear 2 does not move but allows the transmission of force from gear 1. When the motor rotates, gear 1 rotates accordingly, creating force acting on gear 2. Because gear 2 is stationary, this force creates a rotation for the robot’s head, allowing it to rotate left and right around the central axis, thereby mimicking the rotation of a human head looking around. Next, the pitching motion is achieved through the lead screw mechanism, which includes a motor attached to the lead screw shaft through a shaft coupling, creating rotational motion. The lead screw shaft is used to convert the rotational motion of the motor into translational motion and is installed on a flat surface using a rocking mechanism. The mechanism pushes the platform up or down in accordance with the linear motion of the lead screw shaft. Since the platform is designed like a seesaw, the head can move forward or backward. As a result, the robot’s head can nod up and down, simulating a human nodding motion.

**Figure 2:**
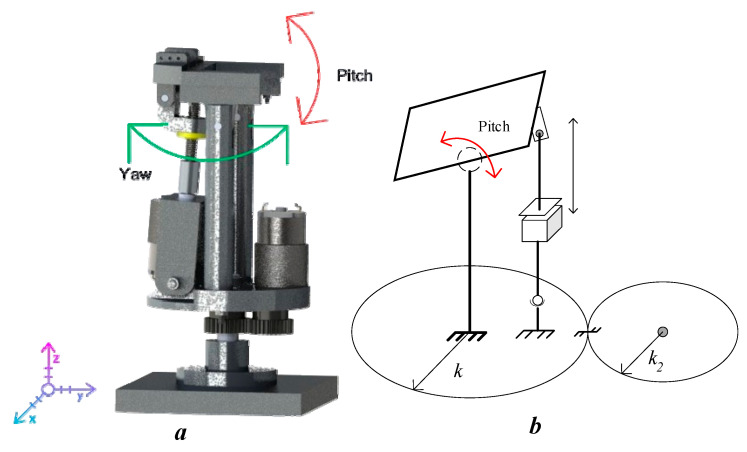
Diagram of the neck mechanism: (**a**) the neck design; (**b**) the kinematic scheme.

The robot neck design integrates these two mechanisms to create a flexible and realistic movement system, helping the robot demonstrate complex movements and interact more effectively. In particular, the rotation and nodding mechanisms operate independently, with the former allowing the robot’s head to rotate left or right, and the latter enabling it to move up or down. In coordinated movement, the combination of these two movements allows the robot head to perform more complex actions. For example, the robot can turn its head to the left while nodding, creating a movement similar to when a human tilts its head in curiosity or when bowing and turning to look around. In short, the neck mechanism with a combination of rotation and nodding motion creates a flexible and natural movement system that mimics the movements of the human neck. This not only enhances the robot’s expressive capabilities but also increases its effectiveness in communication and interaction with the surrounding environment. The mechanism is set up with two degrees of freedom, with the center of gravity placed at the middle of the shaft to reduce pressure on the motors, as shown in Figure 2a. The movements of the neck are shown in Figure 2b, which depicts the kinematic scheme of the robot neck and includes two main angles controlled by the two motors. In this diagram, the *z-axis* is the vertical axis, and the *θ*_1_ (Yaw) angle is the rotation around the *z-axis*. When *θ*_1_ changes, this movement is represented by a rotation around the vertical axis, providing the basis for the robot to look around in the horizontal plane, allowing the robot’s head to perform rotational movements from left to right and vice versa, altering the horizontal viewing direction during the interaction. Similarly, the *x-axis* is the horizontal axis in the kinematic scheme, and the *θ*_2_ (Pitch) angle is the tilt around the *x-axis*. When *θ*_2_ changes, the movement is represented by a rotation around the horizontal axis, facilitating the adjustment of the robot’s viewing angle vertically and allowing the robot to adjust its viewing height. The yaw angle is calculated using Equation (1):

(1) $$\theta_{1}=\frac{k_{2}}{k_{1}}\times \phi$$

where *k*_1_ and *k*_2_ are the radius of gears *Z*_1_ and *Z*_2_, as indicated in Figure 2b; ϕ is the rotation angle of motor 1. The pitch angle is controlled by motor 2 with *θ*_2_ presented by Equation (2):

(2) $$\theta_{2}=K\times \phi_{2}$$

where *K* is the transfer factor; $\phi_{2}$ is the rotation angle of motor 2.

### 3.2. Mouth Mechanism

The human mouth consists of the jaw and lips. The jaw is the main part of the mouth and plays an important role in the process of chewing food [24]. Meanwhile, the lips are the outermost part of the mouth and have many different functions, including protecting the mouth, keeping it moist, participating in speech, and expressing emotions. The human jaw and lips are the most influential in expressing emotions and social interactions. These two parts are not only parts of the body but also powerful tools, helping us express and communicate our emotions, opinions, and mental conditions effectively. The jaw consists of muscle bundles, such as the masseter and temporalis, which not only help us chew food but also exhibit rich emotional expressions. When smiling, the jaw widens and lifts, creating a bright and happy smile. Conversely, in stressful or angry situations, the jaw tenses and grinds the teeth together, showing tension and stress. Despite their small size, lips are an important part of the face and play a large role in expressing emotions. When smiling, the lips widen and lift, creating a bright and cheerful expression. Conversely, when expressing sad or worried emotions, the lips may narrow and pursue, conveying seriousness and insecurity. Accordingly, the parameters of the working space of the human jaw and lips are surveyed to propose the motor to reproduce the movements as shown in Table 5.

The degrees of freedom of the mouth will include the degrees of freedom of the jaw and the degrees of freedom of the lips, allowing for different movements to express emotions. In particular, the human jaw has three degrees of freedom, which include open and close (1 DoF), allowing the lower jaw to open and close to perform actions such as talking and chewing. Sliding anteriorly and posteriorly (1 DoF) allows the lower jaw to move forward and backward, aiding in biting and chewing. Sliding laterally (1 DoF) helps the lower jaw move left and right, which is necessary for even chewing on both sides. The degrees of freedom of the lips enable many flexible movements, with each movement corresponding to a degree of freedom. These include opening and closing (1 DoF), where the upper and lower lips can separate and close, and contraction and expansion (1 DoF), which allow the lips to contract or expand, changing shape to express different emotions. Elevation and descent (2 DoF) enable each lip to be raised or lowered independently, creating different expressions such as smiling or frowning. Finally, angular motion (2 DoF), where each corner of the lips can move at different angles, helps produce complex expressions.

**Figure 3:**
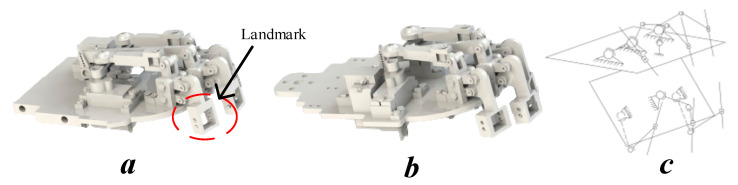
Diagram of the mouth mechanism: (**a**) upper and (**b**) lower lips; (**c**) upper lip mechanism.

In designing a humanoid robot head to create realistic emotions, choosing to use six lip control points instead of eight points is a reasonable and effective decision. Reducing the number of control points helps simplify the design, reduce complexity, and save costs. This also reduces the weight and size of the system, while increasing reliability and making maintenance easier. Although there are fewer control points, six control points can still be enough to create the most basic human expressions such as smiling, grimacing, pursed lips, and open mouth. While eight points can allow for more subtle expressions, six points are capable of producing realistic and natural expressions if controlled properly. Systems with fewer control points are typically less complex and, therefore, less likely to fail. This can increase the reliability of the robot. Fewer control points allow for more efficient control algorithms, focusing on optimizing the software to create the most natural expressions possible. Furthermore, programming and optimizing lip movements become more efficient, focusing on control quality and generating smooth, precise expressions. However, with only six control points, the ability to create complex and delicate expressions can be limited. Secondary emotions, such as a slight smile or wrinkling of the lips, may not be reproduced as accurately as when using eight points. The lack of two control points can reduce the fluidity and naturalness of lip movements, making expressions look more stiff or unnatural. Some specific expressions, such as a sideways lip curl or subtle movements of the corners of the lips, may be difficult or impossible to achieve with just six control points. Nonetheless, with primary emotions, these points can be reproduced completely, and the omission of unnecessary points makes the control and reproduction of movements smoother. The six landmarks are proposed for the lip mechanism as shown in Figure 3 with the upper and lower lips. In Figure 3c, the kinematic diagram clearly outlines the connections and relationships between the motors, lever arms, and the points on the lips, illustrating how the movements are coordinated to achieve realistic lip expressions. Accordingly, the upper lip movement corresponds to the three marked points, and these points are the connection nodes between the motor control mechanisms and the robot’s lips, allowing for up-and-down movement. The lower lip is also represented with three points using a similar mechanism. The movement of the points on the lips through lever arms, along with the concentric joint circles that provide flexible movement, allows the lever arms to rotate in line with motor direction. When a servo operates, it rotates and pulls or pushes the lever arm, resulting in the vertical movement (up or down) of the corresponding control point on the robot’s lips. At the end of each lever arm, the highlighted points are connected to the skin of the robot’s face, ensuring that the lever arm’s movement is directly transmitted to the lip movement. This action mimics the natural movements of the lips and allows the robot to create various facial expressions. The movement parameters and workspaces of the lips are shown in Table 6.

**Figure 4:**
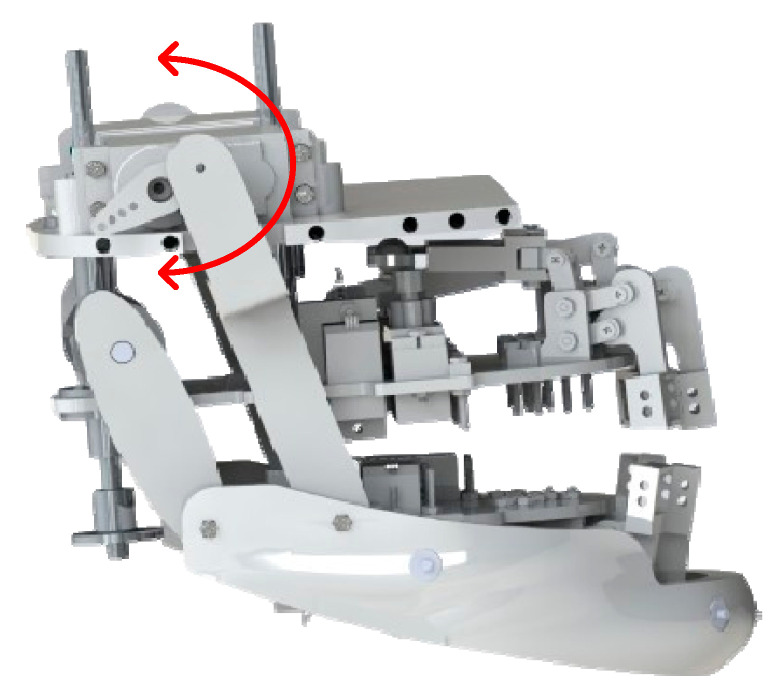
Diagram of the jaw mechanism.

In the robot’s jaw design, the jaw is controlled by two large motors combined with four connecting rods, including two main connecting rods and two rear auxiliary connecting rods as shown in Figure 4. This mechanism allows the jaw to open the mouth at a wider angle, allowing actions such as talking, laughing, or simulated eating to appear natural and realistic. Two large motors provide powerful and stable force to ensure smooth and precise jaw opening and closing movements. When the jaw opens, the connecting rods pull on the lower lip, helping the lower lip move in sync with the jaw, creating a more natural expression. The combination of motors and connecting rods not only enhances the realism of movements but also helps the robot express emotions clearly and effectively, offering a close and vivid communication experience with users. The two rear auxiliary rods play an important role in reducing working space and optimizing jaw movement. They support the primary drive system, helping to distribute force evenly and maintain structural stability as the jaw moves. Thanks to the auxiliary rods, jaw movements become smoother and more precise, minimizing vibrations and increasing the realism of expressions, helping the robot to reproduce actions and emotions more realistically, accurately, and human-like. The coordination between motors and the main and auxiliary rod systems enhances the efficiency and precision of movements and ensures that the robot can express emotions clearly and naturally, providing a vivid and realistic communication experience. The movements of the landmarks of the mouth and jaw mechanism, using a crank-slider mechanism integrated with a four-bar linkage system, are described by the general formula, Formula (3):

(3) $$\theta_{i}=\arccos\left(\frac{l_{1}^{2}-l_{2}^{2}-l_{3}^{2}-l_{4}^{2}+2l_{2}l_{3}}{2l_{2}l_{3}}\right)$$

where *l*_1_ is ground link (fixed); *l*_2_ is crank link; *l*_3_ is coupler link; *l*_4_ is rocker link.

When the motor operates, the rod moves and transmits force to parts of the jaw, contributing to the opening, closing, and movement of the jaw. The motors and connecting rod system provide flexibility and precision in jaw movements. This allows the robot to reproduce emotional expressions realistically and naturally, enhancing its ability to communicate with humans. The robot can maintain consistency and stability in expressions. This makes the robot’s expressions believable and understandable, enhancing interaction and connection with the user. In summary, two important mechanisms in robots to express emotions are the lip and jaw mechanisms. The lip structure, with the support of motors and connecting rods, helps the robot reproduce expressions such as smiling, frowning, and opening the mouth naturally. Meanwhile, the jaw mechanism, controlled by motors and connecting rods, is responsible for actions such as talking and eating. Both mechanisms provide flexibility and precision in the robot’s emotional expression, creating a close and lively communication experience with humans.

### 3.3. Eyes Mechanism

The human eye is one of the most complex and important organs, responsible for vision. It works by capturing light and converting it into nerve signals that the brain can understand. Eye movement is controlled by six peripheral muscles, which help the eye move in a variety of directions, including the superior rectus, which lifts the eye and turns it inward. The inferior rectus muscle lowers the eye and turns it inward [25]. The medial rectus muscle functions to turn the eye inward. The lateral rectus muscle turns the eye outward. The superior oblique muscle turns the eye down and out. The inferior oblique muscle acts to rotate the eye up and out. These muscles work in harmony to perform complex eye movements such as looking up, looking down, looking to the sides, and adjusting vision. This complex control system is operated by nerve signals from the brain, allowing the eyes to move quickly and accurately, giving us the ability to observe and react flexibly to the surrounding environment.

The human eye has a total of three degrees of freedom (DoF), including two main degrees of freedom. Horizontal rotation causes the eyes to move left or right. When rotating the eyes to the right, the lateral rectus muscle of the right eye is responsible for pulling the right eye outward, and the medial rectus muscle rectus of the left eye is responsible for pulling the left eye inward. On the contrary, when turning the eye to the left, the lateral rectus muscle of the left eye is responsible for pulling the left eye outward, and the medial rectus muscle of the right eye is responsible for pulling the right eye inward. These pairs of muscles work in sync with each other to create a smooth movement when turning the eye to the left or right. The external rectus muscle pulls the eye outward, while the medial rectus muscle pulls the eye inward. This precise coordination is controlled by nerve signals from the brain, allowing the eyes to move quickly and accurately. Vertical rotation causes the eyes to move up and down, controlled by the superior rectus muscle, which helps lift the eyes and also helps rotate the eyes inward (intorsion) and move the eyes inward (adduction), while the inferior oblique helps lift the eye and helps rotate the eye outward (extorsion). In contrast, the inferior rectus helps lower the eye and also helps rotate the eye outward (extorsion) and move the eye inward (adduction), in conjunction with the superior oblique muscle, which lowers the eye and helps rotate the eye inward (intorsion). These muscles work in synchrony with each other to produce smooth vertical movements of the eye. The superior rectus and inferior oblique muscles work together to lift the eye, while the inferior rectus and superior oblique muscles work together to lower the eye. Besides, the human eye also has a third degree of freedom, which is axial rotation. This movement involves the eyes rotating around the visual axis, helping to adjust and maintain the stability of the image on the retina when the head is tilted or when the eyes adjust to complex viewing angles. The superior oblique muscle is responsible for turning the eye inward and lowering it, while the inferior oblique muscle helps rotate the eye outward and lift it. Although the axial rotation movement is small and less apparent, it is important to keep the image from turning upside down or tilting, ensuring stable and accurate vision.

The human eyelid has two main degrees of freedom, including elevation and depression movements. The upper eyelid can lift when the eyes are open and lower when the eyes are closed, while the lower eyelid can move slightly up and down. These movements are controlled by three main muscles, including the levator palpebrae superioris, which is responsible for lifting the upper eyelid and helping to open the eyes and is controlled by cranial nerve III (oculomotor nerve). Muller’s muscle also contributes to upper eyelid elevation, especially in maintaining full eye-opening, and is controlled by the autonomic nervous system. In contrast, the orbicularis oculi muscle surrounds the eye, is responsible for lowering the upper eyelid, and helps close the eye, controlling the act of blinking and full eye closure. This muscle is controlled by cranial nerve VII (facial nerve). The movements of the eyeballs and eyelids are described in Table 7. The designs were selected to ensure human-like workspaces. The structures were required to ensure both the workspace and the structure of the movements, as well as the external appearance, were human-like.

**Figure 5:**
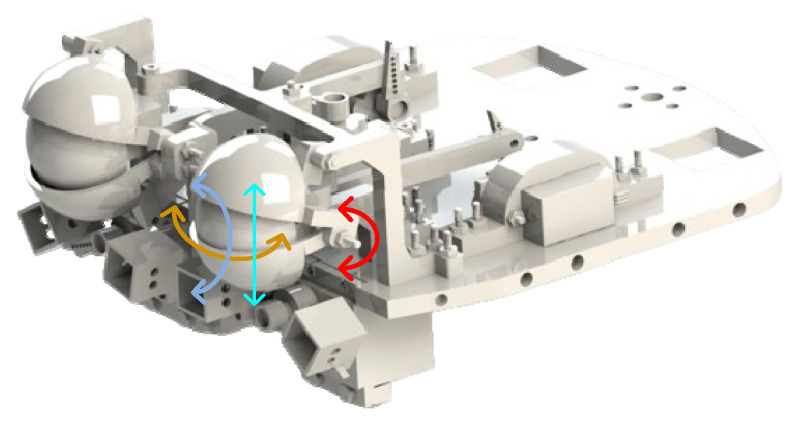
Diagram of the eye mechanisms.

The design of the eye in the robot head consists of a flat transparent mica surface, arranged on the front part of the robot head. This surface is designed to accommodate electronic components such as motors and eye frames. This flat surface can also be made sturdy and precisely machined for installation, and it creates a soft and natural appearance for the robot’s eyes. Next, the bars are arranged vertically or horizontally to secure key components such as eyeballs, eyelids, and motors. Components such as the eyeballs and eyelids are securely attached to these rods, creating a stable and highly precise eye structure. The motors are attached to the rods to control eye movements, including horizontal rotation, vertical rotation, and the opening and closing of the eyelids. The use of motors makes eye movement control flexible and precise and allows the robot to express emotions naturally and realistically. The robot’s eye mechanism uses motors and connecting rods to create synchronized movements between the eyeball and eyelids, creating a realistic and diverse emotional expression. In this way, the robot can move its eyeballs left and right and up and down naturally, just like human eyes. Motors are used to control eye movements, allowing the eyeball to move left, right, up, and down precisely. Connecting rods are connected from the motors to the eyeball and eyelid, ensuring that the movements of the left and right eyes are synchronized with each other. Synchronizing eye movements between the two eyes helps the robot adapt to the surrounding environment and convey emotional expressions naturally. This synchronization creates consistency and reliability in the robot’s emotional expression, helping it interact with humans effectively and engagingly. The mechanism of the eye cluster is shown in Figure 5, with the degrees of freedom annotated by arrows. This structure ensures that the specific requirements for the shape and limitations of the workspaces are met, ensuring that human-like emotions are reproduced. The eye movements are converted from circular motion to translational motion for the eyelids and up–down motion of the eyeball. Hence, these movements are defined by Equation (4):

(4) $$\theta_{i}^{e}=K_{e}\times d_{xy}$$

where $\theta_{i}^{e}$ is the angle of the *i*-th motor; *K_e_* is the transfer factor, for each movement there is a coefficient; *d_xy_* is the distance of the movements in the *x* or *y* axis for the translational movement, and for rotational movement of the eyeballs, *d_xy_* corresponds to the rotation angle.

The mechanism for synchronized horizontal and vertical eye rotation via motors and connecting rods provides many important advantages in terms of ensuring that eye movements, including horizontal and vertical rotation, occur in synchronization with the motors. This provides precise and flexible control over eye movements. The connecting rod effectively drives the motor to the mechanical elements of the eye, ensuring that the eye can move precisely and smoothly. Additionally, the upper eyelid provides a wider range of emotional expression. From emphasizing the eyes to closing or blinking, the robot enables a wide range of situations and emotions flexibly and provides a wider range of emotional expression; they create a friendly and intimate appearance for the robot. In addition, this eye and eyelid mechanism creates natural and consistent eye movements, providing the robot with a wider range of emotional expressions and making the robot more friendly and engaging, because it can “see” and “react” naturally and realistically.

### 3.4. Eyes Brown Mechanism

Human eyebrows are an important part of the face, playing a role not only in protecting the eyes but also in expressing emotions and nonverbal communication. They are important elements in expressing emotions, as they can be raised, lowered, or contracted to express different emotional states such as surprise, anger, sadness, and joy. Eyebrow movements can change the entire look of your face and make it clearer. Eyebrows contribute to the shape and structure of the face. Small changes in the eyebrows, such as shaping or plucking, can dramatically change the appearance of the face. In addition, eyebrows play an important role in nonverbal communication by supplementing or replacing speech, helping the other person better understand the speaker’s emotions and intentions. For example, raised eyebrows can indicate surprise or curiosity, while contracted eyebrows can indicate doubt or disagreement. The human eyebrow has one main degree of freedom, which is the up-and-down movement performed by the frontal muscle group (frontalis muscle). It helps pull the eyebrow up, creating an expression of surprise or interest. Next, the frown muscle (corrugator supercilii muscle) pulls the eyebrow down and toward the center, creating a frowning or concentrated expression. In contrast, the slender muscle (procerus muscle) pulls the middle part of the eyebrow down, often in conjunction with the frown muscle, to create an angry or upset expression. Thanks to the coordination of these muscles, the eyebrows can perform a series of complex and diverse movements, making an important contribution to the expression of human emotions and nonverbal communication. Understanding degrees of freedom and related muscles is also an important basis for designing and programming expressions for humanoid robots. The workspace limits are presented in Table 8.

**Figure 6:**
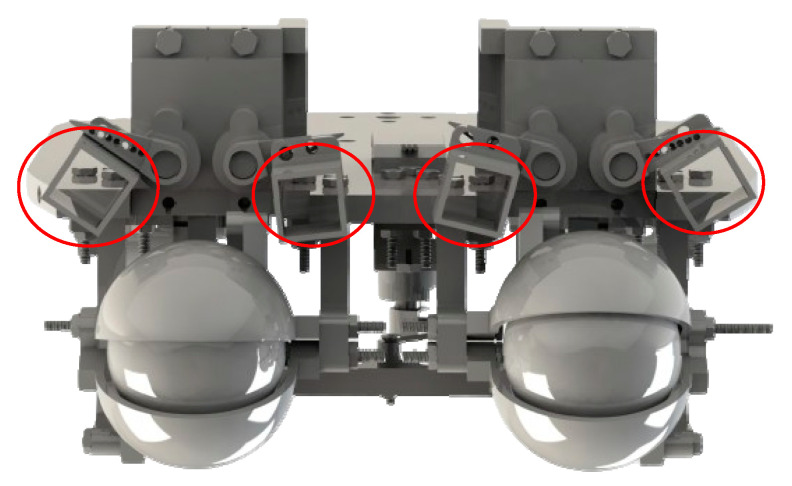
Diagram of the eyebrow mechanisms.

In the eyebrow design, four motors are used to control the movement of four points on the eyebrow, including the start and end points on each side, forming a moving mechanism that adjusts the eyebrow area. Each motor rotates an axis at a certain angle, creating a circular motion. When the motor rotates, the starting point of the eyebrow will move in the direction determined by the linkage design. This movement can be adjusted to rotate clockwise or counterclockwise, depending on the desired direction of the eyebrow movement. This produces a raised or lowered movement of the eyebrow, similar to the natural movement of the human eyebrow. To create natural expressions, the motors at the start and end points need to be controlled synchronously. This ensures that the entire eyebrow moves up or down consistently and naturally. Using the motor’s circular motion to create a circular eyebrow arc is a reasonable and effective method because the operating range of the eyebrow is very limited. This approach helps save space and makes the robot’s structure more gentle in simulating human expressions. Moving landmarks create movements that allow the robot to express basic human-like emotions. The structure is proposed in Figure 6.

## 4. Results and Experiments

**Figure 7:**
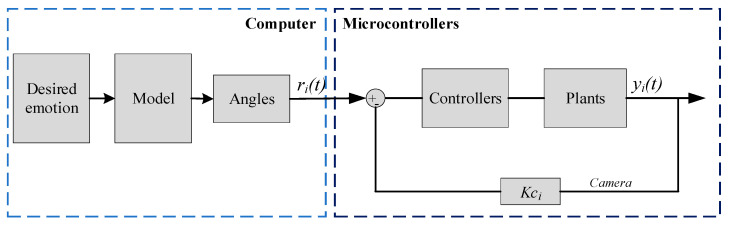
Diagram of the robotic controller.

Figure 7 depicts the control diagram for an actuator with a simple structure and controller. The PID controller [26] is used in this investigation because of its simple operation and calibration. Processing algorithms examine the user’s emotions to determine the rotation angle required for each motor to most accurately mimic the defined emotion. The robot’s image is matched to the movement of landmarks to assess the robot’s appearance. The control system is programmed to replicate and repeat the emotions of the robotic head. The emotions are predicted based on the initial settings of the evaluation process. Based on the fuzzy logic rules set up to give the rotation angles of the motors, fuzzy logic is used to fine-tune to create realistic emotions for the robot, helping the robot avoid the uncanny valley. Closed-loop control is used with feedback signals from the robot image collected from the camera. A gain coefficient of *K_ci_* is calculated to convert into the motor angles; the goal is to bring the errors of *r* and *r_ref_* to *0* in a finite time. The intended emotions are converted into a matrix of angles that must be configured for the robot using Equation (5).

(5) $$r(t)={\left[a_{1},a_{2},\ldots ,a_{n}\right]}^{T}$$

where *a_n_* is the angle of the *n*-th motor.

Let a fuzzy set *V_i_* on the universal set V with *μ_V_*_i_ as the membership function take the form (6). In the landmark recognition model proposed previously, these corner coordinates are fed into the fuzzy model to produce the appropriate rotation angles. The coefficient Kc is fed into the fuzzy model to convert the visual responses into rotation angle errors based on the recognition model. The fuzzy model makes the angle decisions of the motors based on fuzzy membership degrees. The fuzzy system maps the input variables to the output variables based on the expert system consisting of IF–THEN sequences. The fuzzification process is the process of converting the script input and output variables into a set of fuzzy variables. Based on the displacement of the facial landmarks, these are fed into the fuzzification process and calculated. The triangular fuzzy number (TFN) is used to handle uncertainty [27] and is defined as (7) with *V* = (*a*, *b*, *c*). The triangular fuzzy number is given as (8).

(6) $$V_{i}=\{(v,\mu_{V_{i}}(v))|v\in V\},\;where\;\mu_{V_{i}}:V\to [0,1]$$

(7) $$\mu \left(\frac{v_{i}}{V}\right)=\left\{\begin{array}{ll} v_{i}-a/b-a, & a\leq v_{i}\leq b\\ c-v_{i}/c-b, & b\leq v_{i}\leq c\\ 0 & elsewhere \end{array}\right.$$

(8) $$V_{\beta }=[a^{\beta },c^{\beta }]=[a+(b-a)\beta ,\;c-(c-b)\beta ],\;\beta \in [0,1]$$

**Figure 8:**
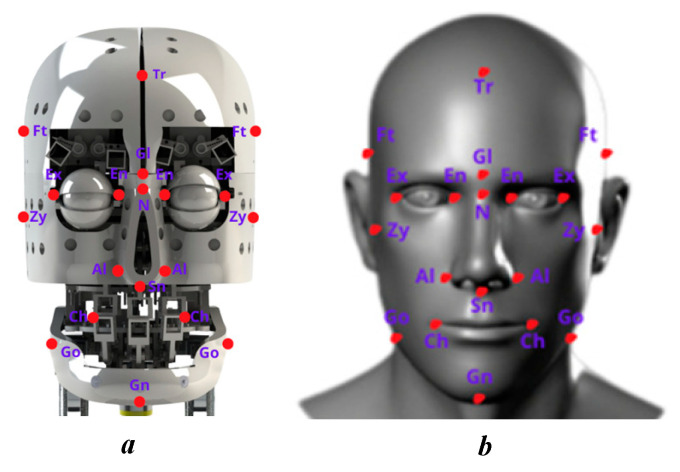
Facial landmarks marked for size measurement: (**a**) facial robot; (**b**) facial human.

To provide a comparative overview and ensure that the robot design can express emotions realistically, the robot size is designed based on human biometric ratios [28]. This helps create a technical specification sheet that closely follows the size and proportions of a real person, thereby improving the robot’s ability to express emotions and interactivity [29]. The neck design for the robot plays a vital role in supporting and maintaining the entire robot head. During the design process, a difference between the size of the robot neck and the human neck may appear due to the technical and functional factors required to ensure the stability and efficiency of the robot. The neck must support the entire robot head, including heavy components such as sensor systems, cameras, and complex motion mechanisms to simulate facial expressions. Therefore, robot necks are often designed with special sizes and structures to ensure stability and durability. The mechanisms are designed in detail to ensure small movements similar to human facial muscles. To accommodate these movements, the degrees of freedom must ensure the implementation of limited angles like humans [30]. The comparison of human and robot movements is presented in Table 9, with the landmarks in the faces of the robot and human shown in Figure 8. Results show that the robot parts can respond to human-like movements, and the limits of all parts are larger than those of humans. The proportion between the human head and the robotic head is calculated in Equation (9).

(9) $$P_{e}=\frac{D_{r}-D_{h}}{D_{h}}\times 100,\;P_{e}=[0,100]$$

where *D_r_* is the size of the robot head; *D_h_* is the average size of Vietnamese head anthropometry. The proportion is only used as a reference to evaluate the deviation between the robotic head and the human head, as it is desired to approach zero to ensure a human-like appearance.

**Figure 9:**
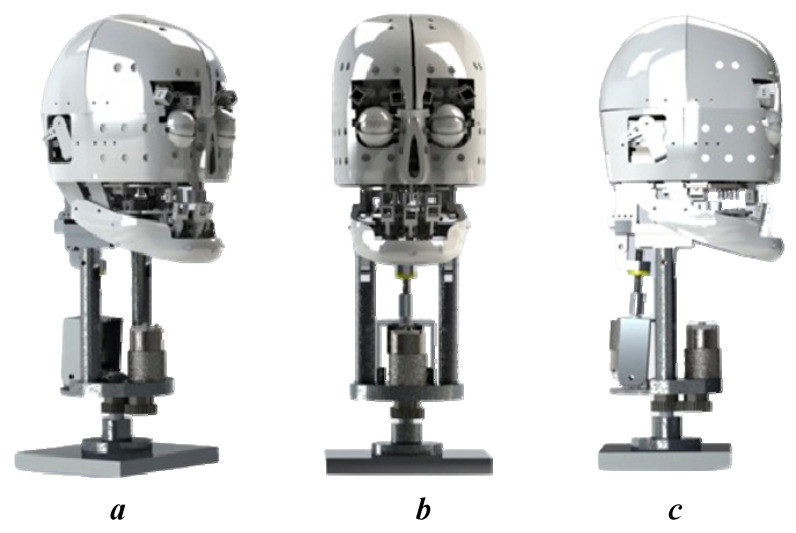
The robot design rendered in software with different views with (**a**) being an isometric view, (**b**) being a front view, and (**c**) being a side view, and back view.

In general, the robot sizes are designed to match the actual human size according to human anthropometric data. Besides, the robot is not designed to copy anyone to ensure identity safety. In the comparison, the ratio between the dimensions of the robot and the real size fluctuates from 0.0425 to 0.1769. This shows that the designs have maintained a reasonable biometric ratio and are consistent with the design and technical structure of the robot. With proportions and sizes approximate to humans, robots can express emotions more realistically. Details such as the distance from the hairline to the most posterior point of the nose, from the hairline to the base of the nose, and from the hairline to the lower chin all play important roles in reproducing facial expressions. The balance and aesthetics of the robot were also considered, as the design tried to keep these proportions within a certain range to create a sense of friendliness and naturalness while communicating. The movements of the robot’s parts correspond to human movements, as demonstrated in Table 10. This ensures that the emotions of the robot are complete and meet the requirements for emotional reconstruction. The design of the humanoid robot is dipected in Figure 9, showing three views that align with human anthropometric dimensions.

**Figure 10:**
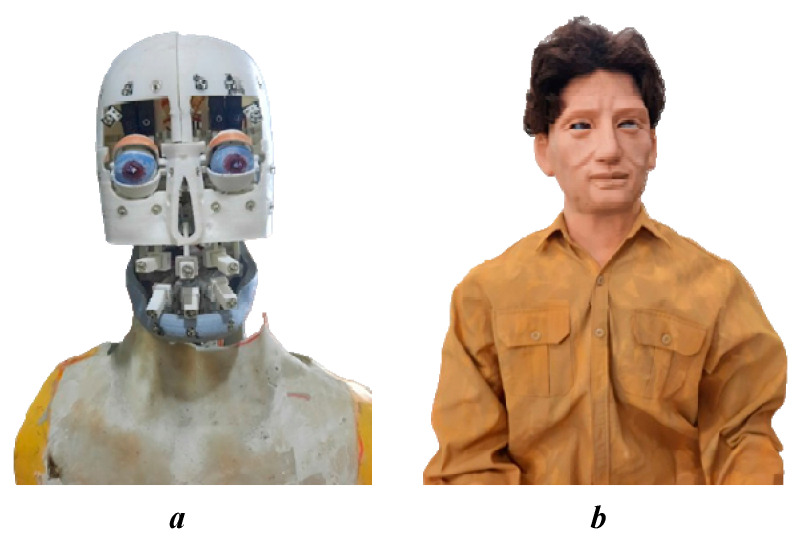
Actual humanoid robot: (**a**) the robot without artificial skin; (**b**) the connection with artificial skin and costumes to give the robot a human-like appearance.

Faces play an essential role in human–robot communication by conveying emotions. As a result, the development of robot appearances has a significant impact on enhancing satisfaction with human interaction. The current robot head design, despite omitting some degrees of freedom, can still display basic emotional states such as happiness, sadness, and surprise. This is a remarkable achievement in the development process, reflecting the focus on creating a robot capable of interacting naturally and expressing emotions effectively. The design has focused on key elements to ensure the robot can still express basic emotions. These include happy states such as smiling, raising the corners of the lips, and opening the eyes wider, which create a feeling of happiness and friendliness; sad states such as lowering the corners of the lips, slightly tilting the head, and reducing the opening of the eyes, which express sadness and sympathy; surprise states such as opening the eyes wide, raising the eyebrows, and opening the mouth wide, which create a surprised expression. The robotic head’s artificial skin is made of silicone rubber and has the same size and form as a human’s head. The robot is built with dimensions based on human anthropometry, as shown in Figure 10. The intricately crafted mechanics guarantee subtle motions akin to those of human facial muscles. The degrees of freedom ensure the application of constrained angles similar to those of humans, allowing for these movements. The experiment included 100 individuals of various ages (average age: 26.68 ± 3.62; 52% female). Each participant was given a questionnaire to evaluate the robot’s emotions; each row included seven options: sadness, happiness, anger, fear, disgust, surprise, and neutral. Participants were asked to choose an emotion they believed the robotic head was exhibiting. The facial expressions that are evaluated through primary emotions are presented in a general way by the confusion matrix shown in Table 11, with an accuracy of 91.50%. This meets the initial goals of robots being able to reproduce human-like emotions.

## 5. Conclusions

In the fast-changing field of robotics and human–robot interaction, integrating emotional intelligence into robots has become critical. This work proposes a thorough design for a robotic head’s biomimetic behavior, with parts and appearance based on Vietnamese anthropometrics to portray human emotions as realistically as possible. The realism of the humanoid robot is demonstrated by the shape and gestures of the muscles on the robot’s face when expressing emotions, thus the data used in the design are thoroughly examined in the dataset. Experimental results through the survey showed that the behavioral simulation rate is up to 94.72%, and the emotional expression rate is 91.50%. The proposed structures are designed to ensure the appropriate size while maintaining sufficient operating space to create realistic emotions. However, in this study, the implementation of mechanical components and materials may be limited in terms of durability because the main component is mainly 3D-printed plastic. In addition, the development direction of the project focuses on increasing the movement points to improve the ability to express emotions, and more experiments with larger sample sets are expected in future work.

## Figures and Tables

**Table 1:** Neoclassical facial proportions.

Name Standard	Measuring Range	Symbol
Upper floor = middle floor = lower floor	Hairline score–Glabella point = Glabella score–point under nose = point under nose–point under the chin	tr-gl =gl-sn = sn-gn
Clear eye angle space = wide nose	Clear eye angle interval = nose wing point interval	en-en = al-al
Clear eye angle space = wide eye	Inner eye base space = eye width	en-en = ex-en
Mouth width = 2/3 of nose width	Oral margin interval = 3/2 aperture point interval	ch-ch = 3/2 * al-al
Standard facial nasal ratio	1/4 of the cheekbones point interval = nose width	1/4 * zy-zy = al-al
n-sn = 0.43 n-gn	Height nose = 0.43 n-gn	n-sn = 0.43 * n-gn

* means the product.

**Table 2:** Muscle bundles and DoFs of body parts.

Body Part	Moving Muscle Bundle	DoF
Neck	Trapezius Sternocleidomastoid Semispinalis Capitis Splenius Capitis	3
Lower Jaw	Masseter Temporalis Medial Pterygoid Lateral Pterygoid	3
Upper Jaw	Not moving	0
Eyes	Superior Rectus Inferior Rectus Medial Rectus Lateral Rectus Superior Oblique Inferior Oblique	3

**Table 3:** Definition of anthropometric measurements collected.

Symbol	Terminology	Definition
Vertical dimensions		
Tr-N	Trichion–Nasion	Hairline point–the most posterior point of the nose on the plane looking inclined to the nose
Tr-Gl	Trichion–Glabella	Hairline point–point on the base of the nose
Tr-Gn	Trichion–Gnathion	Hairline point–lower chin point
Gl-Sn	Glabella–Subnasale	Point on the root of the nose–point under the nose
N-Sn	Nasion–Subnasale	Nasal root point–subnasal point
N-Gn	Nasion–Gnathion	The deepest point of the nose on the plane looks inclined to the nose–lower chin point
Sn-Gn	Subnasale–Gnathion	Subnasal point–lower chin point
Horizontal dimensions		
En-En	Endocanthion–Endocanthion	Corners of the eyes on the left and right
Ex-En	Exocanthion–Exocanthion	Inner–outer eye corners
Go-Go	Gonion–Gonion	The farthest distance between the two angles of the left and right jaws
Zy-Zy	Zygion–Zygion	Cheekbone point interval
Ch-Ch	Chinion–Chinion	Left–right corner of the mouth
Al-Al	Alare–Alare	The outermost point in the left canards–the outermost point in the right canards
Ft-Ft	Frontotemporal–Frontotemporal	Distance between two temple points

**Table 4:** Movements and workspaces of the neck.

Movement	DoF	Workspace
Nod	1	Tilt upward by 45–70 degrees Bow 50–60 degrees
Tilt	1	Average per side: 40–45 degrees
Rotation	1	Average per side: 60–80 degrees

**Table 5:** Movements and workspaces of the jaw and lips.

Movement	DoF	Workspace
Jaw	3	40–60 degrees
Lips	6	15–20 degrees

**Table 6:** Movements and workspaces of the lips.

Movement	DoF	Workspace
Upper lips	3	15–20 degrees
Lower lips	3	15–20 degrees

**Table 7:** Movements and workspaces of the eyeballs and eyelids.

	Movement	DoF	Workspace
Eyeballs	Horizontal rotation	1	Rotate left by 45–55 degrees Rotate right by 45–55 degrees
	Vertical rotation	1	Rotate up by 45–60 degrees Rotate down by 50–60 degrees
Eyelids	Upper eyelid	1	20–30 degrees
	Lower eyelid	1	2–5 degrees

**Table 8:** Movements and workspaces of the eyebrow.

Eyebrow Movement	Workspace
Lift	Move up about 1–2 cm from the natural position
Lower	Move up about 0.5–1 cm from the natural position

**Table 9:** Dimensions used to design the robot and proportion.

Order	Symbol	Robot	Human	Proportion
1	*Tr-N*	85.17	78.89	7.96
2	*Tr-Gl*	74.49	63.71	16.92
3	*Tr-Gn*	221.95	194.78	13.95
4	*Gl-Sn*	70.02	64.08	9.27
5	*N-Sn*	59.34	50.50	17.51
6	*N-Gn*	137.03	116.43	17.69
7	*Sn-Gn*	77.44	65.14	18.88
8	*Ft-Ft*	148.32	142.27	4.25
9	*Zy-Zy*	149.15	147.15	1.36
10	*Go-Go*	113.25	126.94	10.78
11	*Al-Al*	37.31	42.71	12.64
12	*En-En*	32.97	37.85	12.89
13	*En-Ex*	34.04	35.98	5.39
14	*Ch-Ch*	53.09	64.07	17.14

**Table 10:** Comparison of the workspaces of parts of robots and humans.

Order	Part		Robot	Human
1	Neck	Pitch	60°	57.5°
		Yaw	180°	70°
2	Jaw		52°	36°
3	Lip	Upper and lower	20 mm	17 mm
		Front lip edge movement	18 mm	11 mm
4	Eye	Upper–lower	110°	70°
		Left–right	160°	70°
5	Eyelid	Upper	65°	30°
		Lower	65°	22°
6	Eyebrow		15 mm	10 mm

**Table 11:** Confusion matrix of emotion reconstruction on the robot’s face.

	Surprised	Happy	Disgust	Sad	Anger	Fear
Surprised	90	2	0	0	0	4
Happy	3	92	2	1	4	0
Disgust	0	4	91	0	0	0
Sad	0	1	0	92	0	2
Anger	1	1	5	0	94	3
Fear	4	0	1	5	2	90
Error	2	0	1	2	0	1
Accuracy	0.9	0.92	0.91	0.92	0.94	0.9

## Data Availability

All data generated or analyzed during this study are included in this published article.
